# Terahertz Imaging of Thin Film Layers with Matched Field Processing

**DOI:** 10.3390/s18103547

**Published:** 2018-10-19

**Authors:** Scott Schecklman, Lisa M. Zurk

**Affiliations:** 1 Electrical and Computer Engineering Department, Portland State University, Portland, OR 97201, USA; zurkl@pdx.edu; 2 Applied Physics Laboratory, University of Washington, Seattle, WA 98105, USA

**Keywords:** terahertz, non-destructive evaluation, tomography, super-resolution, matched field processing, covariance matrix, Bartlett, minimum variance, ambiguity

## Abstract

Terahertz (THz) time of flight (TOF) tomography systems offer a new measurement modality for non-destructive evaluation (NDE) of the subsurface layers of protective coatings and/or laminated composite materials for industrial, security and biomedical applications. However, for *thin film* samples, the time-of-flight within a layer is less than the duration of the THz pulse and consequently there is insufficient range resolution for NDE of the sample under test. In this paper, matched field processing (MFP) techniques are applied to thickness estimation in THz TOF tomography applications, and these methods are demonstrated by using measured THz spectra to estimate the the thicknesses of a thin air gap and its depth below the surface. MFP methods have been developed over several decades in the underwater acoustics community for model-based inversion of geo-acoustic parameters. It is expected that this research will provide an important link for THz researchers to access and apply the robust methods available in the MFP literature.

## 1. Introduction

In recent years, Terahertz (THz) tomography has emerged as a promising new technology with NDE applications in a variety of fields, including industrial manufacturing, security screening and medical imaging [1,2]. THz time domain spectroscopy (TDS) systems provide high dynamic range over a relatively wide bandwidth in the far infrared portion of the electromagnetic spectrum (0.3–3.0 THz) and are therefore uniquely suited for investigation of the sub-surface layers of dielectric packaging and coating materials. Some examples of THz NDE applications that have been demonstrated recently include analysis of the coating layers of pharmaceutical tablets [3,4,5], paint layers in the automotive industry [2,6,7,8]. In addition, THz NDE is being explored for lightweight composite materials used in construction [9], aerospace [10] and personal armored materials, such as bullet-proof vests [11]. Potential biomedical applications include NDE of the surface layers of the skin and teeth [2].

Many samples of interest for these applications contain a subsurface layer with high attenuation that limits the THz measurement configuration to a reflection geometry. Often the dielectric materials of the packaging and/or surface coating layers exhibit relatively low loss, and abrupt changes in the refractive index at the layer boundaries can be observed as a pulse train (A-scan) of reflected THz pulses. For *thick* layered media, signal processing methods such as deconvolution or matched filtering can be used to approximate the impulse response of the sample and the propagation delay between impulse arrivals can be multiplied by the group velocity to compute the layer thickness [2,12]. Thus, THz time of flight (TOF) tomography uses the time difference of arrival (TDOA) between the THz pulse train that is reflected from the material boundaries to generate an image of the subsurface features within a sample [2].

However, for *thin films*, the time delay between THz echo pulses from the upper and lower layer boundaries is less than the duration of the THz pulse, and there is insufficient axial resolution for NDE of the sample under test. Various approaches have been considered to estimate film thickness [13], and in recent years model-based parameter estimation has emerged as a promising approach for THz NDE of thin films [11,14,15,16,17,18,19]. The general approach is to use an objective function to compare the measured THz waveform (including overlapping pulses) and each one of a set of simulated THz waveforms that are generated by a propagation model. Much of the recent THz NDE literature has focused on propagation modeling methods and advanced search algorithms to efficiently identify the best estimate(s) for the parameter(s) [16,17,18,19].

THz tomography is still a relatively new area of research and physics-based signal processing methods are currently being explored. In this paper, MFP is introduced as a potential method for thickness estimation of both thick and thin layers in THz tomography applications. MFP has been used for decades in the underwater acoustic signal processing community, but to the authors’ knowledge has not been applied to THz tomography until now.

### 1.1. Matched Field Processing (MFP)

MFP is a robust model-based parameter estimation approach that developed primarily in the underwater ocean acoustics community during the 1980s and early 1990s [20,21]. Historically, MFP has been used for sound source localization when simple plane wave beamforming techniques could not account for complicated acoustic propagation within the dynamic ocean environment [20,21,22]. Typically, environmental parameters are measured independently or assumed to be known and the goal of MFP is to use data recorded with an array of hydrophone receivers to estimate the position of the sound source (i.e., its range, depth and sometimes azimuth), relative to the receiver position.

A logical extension for these techniques has been to record acoustic data with a known sound source position and estimate the environmental parameters between the source and retriever(s). The use of MFP for estimation of environmental properties is sometimes referred to as matched field tomography in the literature [20,23]. One important example of matched field tomography is the estimation of geo-acoustic properties of the sea floor, including thickness of sediment layers as well as the sound speed and attenuation within each layer.

MFP techniques were developed to work in complex remote sensing applications that involve in situ sensor arrays moving in noisy and dynamically changing environments, and can be characterized by several processing components [20,22,23]. Rather than attempting to match the modeled fields directly to the measured fields, the covariance of the measured field is often used. The covariance accounts for relative differences between the discretely sampled field measurements, and is therefore less sensitive to mis-matches due to sensor motion and/or changes in the data collection environment [20,24]. The most common objective functions in the MFP literature include the Bartett and minimum variance (MV) processors. Often, a uniformly spaced grid search is used to identify a global maximum in the objective function data, and results of the search are organized to plot a 2D ambiguity surface which can be used to examine the precision and accuracy of the results. These processing techniques are discussed in further detail in Section 2.

In the late 1990s, MFP was applied to electromagnetics for the radar systems locating airborne vehicles within the layers of the troposphere [25]. MFP remains an active field of study in the underwater acoustics community [22]. However, to the author’s knowledge, MFP techniques have not been applied to THz tomography until now.

### 1.2. Applying MFP Techniques to THz Tomography

The primary aim of this work was to apply the MFP techniques that have been developed in the underwater ocean acoustics community to THz tomography applications. Here, the objective was to use data recorded with a THz sensor to estimate the thickness of subsurface layers of a dielectric material, which is similar to the ocean acoustic applications of matched field tomography to estimate sediment layers.

In underwater acoustics applications, the sources are typically narrow-band and broadband sources are usually temporally incoherent. Therefore, conventional MFP typically exploits only the spatial coherence of the field (at a single frequency) as measured by an array of sensors after performing a Discrete Fourier Transform (DFT) on the data recorded by each sensor [20,21,22]. However, when the source is temporally coherent across a broad band of frequencies, then the temporal coherence may also be exploited. Work by Tolstoy [21], Michalopoulou [26,27], Siderius [28] and others has shown that both the spatial and temporal coherence of the field can be accounted for by concatenating the discrete frequency spectra from each of the sensors in an array. A summary of MFP techniques that exploit both spatial and temporal coherence has been given by Dosso [29].

The following section outlines formulations for applying the mathematical notation and methods typically found in the MFP literature to the electric field measurements that are often recorded by THz sensors. THz-TDS systems generally consist of only one sensor due to the complications involved in coherently combining data from multiple receivers in real time. Therefore, in this paper, MFP is used to exploit the temporal coherence of the THz spectrum from a single THz sensor to extract thickness information from thin film materials. However, the techniques presented here could also be extended to multiple sensor arrays (such as synthetic aperture arrays) using mathematical approaches in the MFP literature [29]. THz synthetic aperture imaging can be performed with a monostatic THz emitter/sensor pair that is raster scanned above the sample under test [30,31,32,33].

Section 3 presents ambiguity images for several THz tomography experiments performed for NDE of an air film layer embedded within a polycarbonate background. The temporal coherence of the THz waveform is expressed in terms of the covariance of the THz spectrum. The covariance matrix of the measured field is then compared with a family of modeled fields using two of the most popular processors (objective functions) in the MFP literature: the Bartlett processor and the MV processor. The precision of the estimates is evaluated with images of the ambiguity surfaces. In the axial dimension, super-resolution is demonstrated by accurately estimating the thickness of a thin film of air embedded within a polymer (polycarbonate) background. A discussion of the significance of the results with suggestions for possible future extensions is provided in Section 4. It is expected that this research will provide an important link between THz NDE applications and some of the advanced physics-based signal processing methods that have already been developed in the MFP literature over the past several decades.

## 2. Methodology

This section reviews the conventional method of estimating the thickness of layered media from THz TDS measurements. The axial resolution limit is discussed and MFP is introduced as a new approach that can be used for both thick and thin layers. MFP is a model-based parameter estimation technique that exploits a priori knowledge of the measurement geometry and the sample configuration to provide super-resolution tomography data.

### 2.1. Conventional THz TOF Processing

THz TOF tomography systems are typically composed of a single source and receiver due to the complexities associated with coherently combining multiple receivers. Often, the measurement configuration is oriented in a reflection geometry because many of the samples of interest contain a subsurface layer with high attenuation. Thus, the THz source pulse is incident on the sample under test and reflections from the layer boundaries are observed as a pulse train (A-scan) of reflected THz pulses recorded at the receiver. If the index of refraction within a given layer is known, then time difference of arrival (TDOA) between THz pulses that are reflected from the two boundaries can be used to calculate the thickness of the layer. The axial range (or depth) resolution of the THz pulse is

(1) $$d_{min}=\frac{\tau v_{g}}{2}$$

where τ is the duration of the THz pulse and $v_{g}$ is the group velocity of the pulse within the layer. The group velocity is $v_{g}=c/n_{g}$, where $n_{g}$ is the group (refractive) index and $c=3\times 10^{8}$ m/s is the speed of light in vacuum. Thus, the axial resolution depends on the material within the layer boundaries and also on the duration of THz source pulse.

For a layer with thickness, $d<d_{min}$, the THz pulse reflecting from the back surface of the layer will overlap with the pulse reflecting from the front surface, and estimation of the thickness from the TDOA of the pulses is not possible. Model-based parameter estimation can be used to estimate the thickness of thin film layers. The remainder of this section provides the theoretical background to apply MFP techniques to estimate the thickness of thin films with THz-TDS pulses.

### 2.2. Mathematical Model for Terahertz Measurement Data

For a THz-TDS system, the received time-domain waveform can be expressed as a function of time, *t*, and the parameters of interest for the sample under test. In general, there can be many parameters, $p_{1},p_{2},\ldots$, and therefore it is convenient to denote them as a single vector, $a={\left[p_{1},p_{2},p_{3},\ldots \right]}^{T}$, where the superscript *T* represents the matrix transpose operation. The *true* parameters of the sample are denoted $a_{T}$, and MFP is used to compare measured and modeled field data using an objective function to arrive at a best estimate the parameter vector, denoted $\hat{a}$.

The spectrum of the received electric field, $R(f,a_{T})$, can be expressed as the convolution of the source spectrum, D(f), with the transfer function of the sample, $H_{s}\left(f,a_{T}\right)$, and the addition of measurement noise, N(f). Thus, the received spectrum is modeled as

(2) $$R\left(f,a_{T}\right)=D\left(f\right)H_{s}\left(f,a_{T}\right)+N\left(f\right),$$

where D(f) represents the convolution of the instrument response and the transfer functions that accounts for propagation through the various components of the THz measurement system and the background medium, excluding the sample under test.

The unknown system variables in D(f) can be accounted for with a reference waveform that is collected with a known sample having a transfer function, $H_{s}\left(f\right)$, that does not depend on the parameter vector. Many measurements of the reference waveform are collected and the received spectrum is averaged over a long integration time to reduce the effects of measurement noise. This longer integration time is acceptable in THz NDE applications because, unlike the sample measurements, this reference only needs to be recorded once for a given measurement configuration.

Thus, after averaging many measurements, the mean received field can be approximated as

(3) $$R_{ref}\left(f,a_{T}\right)\approx D\left(f\right)H_{s}\left(f,a_{T}\right),$$

For a THz system configured in reflection mode, the reference signal is measured with the sample replaced by a mirror positioned as close as possible to same position as the surface of the sample. Then, the transfer function of the sample is simply, $H_{s}\left(f\right)\approx -1$. Therefore, $D\left(f\right)\approx -R_{ref}\left(f\right)$, and the received spectrum for a given layered media sample can finally be expressed as

(4) $$R\left(f,a_{T}\right)=-R_{ref}\left(f\right)H_{s}\left(f,a_{T}\right)+N\left(f\right).$$

### 2.3. Generating THz Replica Spectra

The MFP algorithm requires a forward propagation model to generate replicas of the THz spectrum that can be later compared with the measured THz spectrum by an objective function. Various propagation models could be used within MFP depending on the geometric configuration of the source, sample and sensor(s). A propagation model for the transfer function, $H_{s}\left(f\right)$, for a parallel stack of layered media is presented in Appendix A.

The transfer function in Equation (A1) can be parameterized in terms of variables within the model. For example, $H_{s}\left(f,a\right)$, where $a={\left[d_{1},d_{2},\ldots d_{Q}\right]}^{T}$, with the thickness of each layer, $d_{q}$, used in Equation (A5) of the model. Similarly, the refractive index in Equation (A4) could be further parameterized using the Lorentz model, Drude model, and/or various effective media models that can account for random scattering within the layer(s) using an effective index of refraction.

Thus, the model presented in this section can be used to generate a set of simulated *replica* fields,

(5) $$R_{r}\left(f,a\right)=D\left(f\right)H_{s}\left(f,a\right),$$

where the subscript *r* denotes replica field and D(f) is obtained from a reference measurement as discussed above.

In practice, the measured field is discretely sampled by the THz measurement system, with the total of *L* time samples in the measured waveform. Then, the Fourier transforms discussed in the previous section can be realized as a discrete Fourier transform (DFT) that results in discrete frequency bins. The discrete spectrum can then be truncated to a bandwidth of *L* frequency bins within which there is sufficient signal-to-noise ratio (SNR). Thus, the discrete spectrum of the received spectrum can be denoted as a vector, $R(a_{T})$, of length *L*. Note, the dependence on frequency, *f*, is assumed in the vector notation.

Similarly, the replica fields that are generated with Equation (5) can be modeled at the same discrete frequencies as the measured field (Equation (4) for direct comparison using an objective function. Thus, the replica fields can also be expressed as vectors, $R_{r}\left(a\right)$, of length *L*.

It is important to note that the propagation model in Appendix A, inherently includes an infinite number of multipath reflections in each layer [34], and under-sampling the field in the frequency domain can result in aliased multipath arrivals in the modeled field data. Therefore, the modeled fields should be generated at small frequency intervals, and then an inverse transform should be performed to project the modeled fields into the time domain where they can be truncated at the same duration as the measured fields. Finally, a DFT is performed on the truncated replica waveforms to create the replica spectra, $R_{r}\left(a\right)$. These replica spectra are then truncated to include the same bandwidth as the the measured spectrum, $R(a_{T})$.

Finally, a normalized weight vector, w(a), is created from the replica field,

(6) $$w\left(a\right)=\frac{R_{r}\left(a\right)}{\left|R_{r}\left(a\right)\right|}.$$

### 2.4. Sample Covariance Matrix

The weight vector could be compared with the measured field vector, but it is more common in the MFP literature to use the covariance of the measured field. The covariance accounts for relative differences between the discretely sampled field measurements, and is therefore less sensitive to mis-matches due to sensor motion and/or changes in the data collection environment [20,24]. The covariance matrix of the measured field is approximated as

(7) $$\hat{K}\left(a_{T}\right)\approx \frac{1}{Z}\sum_{z=1}^{Z}\left(R_{z}\left(a_{T}\right)\times R_{z}^{H}\left(a_{T}\right)\right),$$

where $R_{z}\left(a_{T}\right)$ is the measured field for each of the *Z* snapshots, and the superscript *H* indicates Hermetian transpose.

The covariance is a square [L×L] matrix composed of complex numbers. Each element of the covariance matrix contains information about the relationship of one discretely sampled bin with one of the other bins. Therefore, the elements along the diagonal of the covariance matrix are the self-terms, and the covariance matrix is complex symmetric along the diagonal. Elements of $\hat{K}\left(a_{T}\right)$ with large covariance values indicate a stronger relationship between the two measurement bins than an element with a covariance that is near zero.

Ideally, each of the rows (or columns) of the covariance matrix should be linearly independent, i.e., the covariance matrix should be full rank. A rule of thumb is to ensure that the total number of snapshots is greater than the length of the measured data vector, i.e., Z>L. This is sometimes used as a lower limit for the total number of snapshots required.

### 2.5. Objective Functions

In the MFP literature, an objective function, sometimes called a cost function or “processor”, is used to compare each of the replicas, with the measurement data, and then a search algorithm is used to find the replica field that is the best match to the measurement. In this section, two of the most popular processors in the MFP literature, the Bartlett processor and the MV processor, are briefly discussed.

In addition to the Bartlett processor and MV processor, various other processors have also been used [20,21,22]. The trade-offs between various other objective functions is beyond the scope of this paper, but can be found in the MFP literature [20,21,22].

#### 2.5.1. Bartlett Processor

The Bartlett processor is perhaps the most widely used processor in the MFP literature [20,21,22]. It can be expressed as the average of the projection of the measured data vectors on the normalized replica vectors [20],

(8) $$P_{B}\left(a\right)=\frac{1}{Z}\sum_{z=1}^{Z}{\left|w^{H}\left(a\right)R_{z}\left(a_{T}\right)\right|}^{2},$$

which can be computed in with the covariance matrix as follows:

(9) $$P_{B}\left(a\right)=w^{H}\left(a\right)\hat{K}\left(a_{T}\right)w\left(a\right).$$

The trial parameter vector, a, that results in a global maximum in $P_{B}\left(a\right)$ is regarded as the best estimate for the parameter(s) and is denoted ${\hat{a}}_{B}$.

The Bartlett processor is robust to modeling inaccuracies and is relatively straight-forward to implement [21].

#### 2.5.2. Minimum Variance (MV) Processor

The MV processor (also known as Capon processor) [20,21,22] is an adaptive processor that suppresses ambiguities. It can be expressed as

(10) $$P_{MV}\left(\hat{a}\right)=\frac{1}{w^{H}\left(\hat{a}\right){\hat{K}}^{-1}\left(a_{T}\right)w\left(\hat{a}\right)}.$$

The trial parameter vector, a, that results in a global maximum in $P_{MV}\left(a\right)$ is regarded as the best estimate for the parameter(s) and is denoted ${\hat{a}}_{\mathrm{MV}}$.

The MV processor is capable of providing very precise results (narrow ambiguity) when the simulation inputs are well-known, but may provide inaccurate results when they are not.

It is important to note that the MV processor requires the inverse of the sample covariance matrix, ${\hat{K}}^{-1}$ to be computed. This can be problematic if the sample covariance matrix is not full rank, which can happen when there is an insufficient number of snapshots included in the averaging performed in Equation (7). If there is insufficient time available to collect additional snapshots, then a small quantity can be added to the diagonal elements of the covariance matrix, which is often referred to as diagonal loading. More details about diagonal loading can be found in Chapter 3 of [21].

### 2.6. Ambiguity Surfaces

The parameter(s) that results in the optimum output from the objective function is identified as the best estimate of the measured variable. There may be multiple local peaks in the ambiguity surface and it is therefore important that replicas are generated for the entire region of feasible values of each of the free variables in the parameter vector, a, and that the search algorithm identifies the global maximum in the resulting ambiguity data.

In addition to identifying the parameter values that created the global peak in the ambiguity surface, analyzing the ambiguity in the neighborhood of the peak value give some indication of the confidence in (or precision of) the estimated parameter values. Therefore, plots of the ambiguity surfaces often appear in the MFP literature [20,21,22]. If there is only one free parameter in the MFP algorithm, then the ambiguity surface is simply a line plot. If there are two free parameters, the ambiguity surface is generally shown as a 2D image. For MFP with more than two dimensions, it is possible to explore the multi-dimensional ambiguity space by selecting the peak value in all but two of the dimensions and then plotting the 2D ambiguity surface vs. the two remaining dimensions.

### 2.7. Accuracy Limitations

The previous discussion in this section outlined the MFP methodology for using measured THz data and a set of modeled replicas to estimate unknown parameters (such as layer thickness) for a sample under test. In addition, it is sometimes desirable to also determine the minimum accuracy that could be expected for a given measurement system and/or sample.

The limitations on the accuracy of the parameter estimation can be evaluated using the Cramer–Rao lower bounds, which are expressed in terms of the elements of the inverse of the Fischer information matrix [20,35,36]. The elements of the Fischer information matrix are functions of the true values of the parameters, and therefore the minimum achievable accuracy depends on the particular sample under test. Formulation of the Cramer–Rao lower bounds for THz NDE applications is beyond the scope of this paper. Resources for further study of accuracy limitations in parameter estimation can be found in the literature [20,35,36].

## 3. Results

This section describes THz experiments that use the MFP approach presented in the previous section for NDE of a polymer that contains an air film below the surface. Thus, the examples considered here could be representative of industrial NDE applications which require detection and evaluation of defects (e.g., air bubbles) below the surface of the sample under test.

### 3.1. Terahertz Measurement System

**Figure 1:**
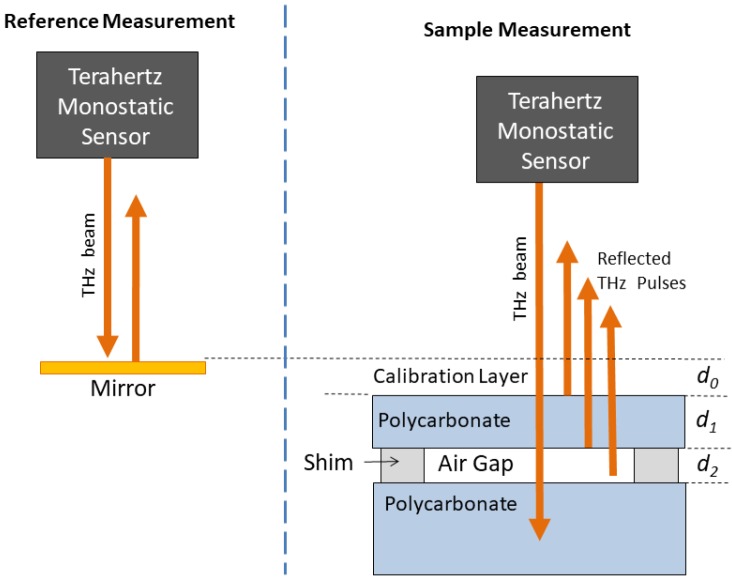
Illustration of the THz NDE experiment configuration (not to scale). (**Left**) Measurement configuration for the THz reference, which is used to approximate the THz source signal in MFP processing. (**Right**) Measurement configuration for the layered sample under test. A calibration layer (air) with unknown thickness, $d_{0}$, accounts for the offset distance between the reference mirror and the surface of the sample. A shim (Scotch^®^ Removable Double Sided Tape) located a distance of $d_{1}$ below the sample surface creates an air gap with thickness, $d_{2}$. THz MFP is used to estimate the thicknesses of all three layers ($d_{0},d_{1},$ and $d_{2}$), simultaneously.

The experiments discussed here were performed in the Northwest Electromagnetics and Acoustics Research Laboratory (NEAR-Lab) at Portland State University (PSU) with a Picometrix T-Ray 4000 THz time-domain spectroscopy (TDS) system from Advanced Photonics, Inc. Details about the THz-TDS system are available in [12]. The THz-TDS system was configured for monostatic measurements at normal incidence, as illustrated in Figure 1.

All of the experimental data presented in this paper were collected using a collinear measurement head that contains transmit and receive modules joined with a duplexer into a single unit for monostatic measurements. The collinear head was fit with a collimating lens and oriented to provide a THz beam at normal incidence on an adjustable sample stage. The stage was mounted to an optical workbench and adjusted in three dimensions (yaw, pitch, and roll) so that the sample media was perpendicular to the THz beam.

As discussed in the previous section, THz MFP in reflection configuration requires the THz source signal to be approximated with a reference measurement from a mirror. The left side of Figure 1 shows the configuration for the THz reference measurement from a gold mirror. A total of 10,000 reference waveforms were averaged to maximize the SNR. After performing a DFT, the reference spectrum could be used to approximate the source spectrum, D(f), needed to generate the replica spectra, $R_{r}\left(f,a\right)$, in Equation (5).

### 3.2. Layered Media Samples

The right side of Figure 1 illustrates the measurement configuration for the sample under test, which consisted of a small air gap between two layers of polycarbonate. The polycarbonate layers were homogeneous with smooth parallel surfaces, making them ideal for a variety of layer configurations for THz laboratory measurements. Thus, the samples considered here are representative of NDE applications in which it is desirable to know the thickness of a defect, e.g., an air bubble ($d_{2}$), and its depth below the surface ($d_{1}$).

In practice, there is always a small offset distance between the surface of the sample under test and the surface of the mirror during the reference measurement. This small offset can be accounted for with MFP by including an additional thin layer of air above the sample’s surface when generating replica spectra with the propagation model [16]. In the remainder of this paper, this hypothetical layer will be referred to as the *calibration* layer, denoted $d_{0}$. The air gap between two layers of polycarbonate was created using a shim (Scotch^®^ Removable Double Sided Tape 667, Cat. 238) with a thickness of 2.4 mils (61 μm), per the manufacturer.

The depth of the air gap below the polycarbonate surface is $d_{1}$. Four different samples thicknesses for layer $d_{1}$ were tested using polycarbonate film samples obtained from Tap Plastics, Inc. (Fremont, CA, USA) with thicknesses of 10,15,20 and 30 mils (1 mil = 1/1000 inch = 25.4 μm). Each of the samples was measured with a digital Vernier caliper with 10 μm resolution and 20 μm accuracy. The average of 10 measurements for each sample film is recorded in Table 1.

A THz measurement was performed in transmission configuration with a single layer of polycarbonate (Sample A in Table 1) to extract the material properties of polycarbonate. The THz TDS signal processing methods for extraction of material properties from solid materials are already documented in the literature [2,37] and therefore are not discussed in detail here. The resulting complex index of refraction were consistent are similar to the data for polycarbonate shown elsewhere in the literature [2,38]. The real part of the refractive index is approximately 1.6 across the 0.1–1.3 THz band, and the extinction coefficient increases monotonically with frequency (with a value of about 0.018 at 0.5 THz and 0.025 at 1.0 THz).

### 3.3. Measurement Data Processing

The monostatic THz sensor configuration shown in Figure 1 was used to record THz waveforms above Sample A in Table 1. In total, 300 waveforms were each recorded at a single position with minimal measurement integration time (10 ms/waveform). The waveforms were truncated within a time window with duration of 60 ps surrounding the reflected pulses from the surface and the air gap below the surface.

**Figure 2:**
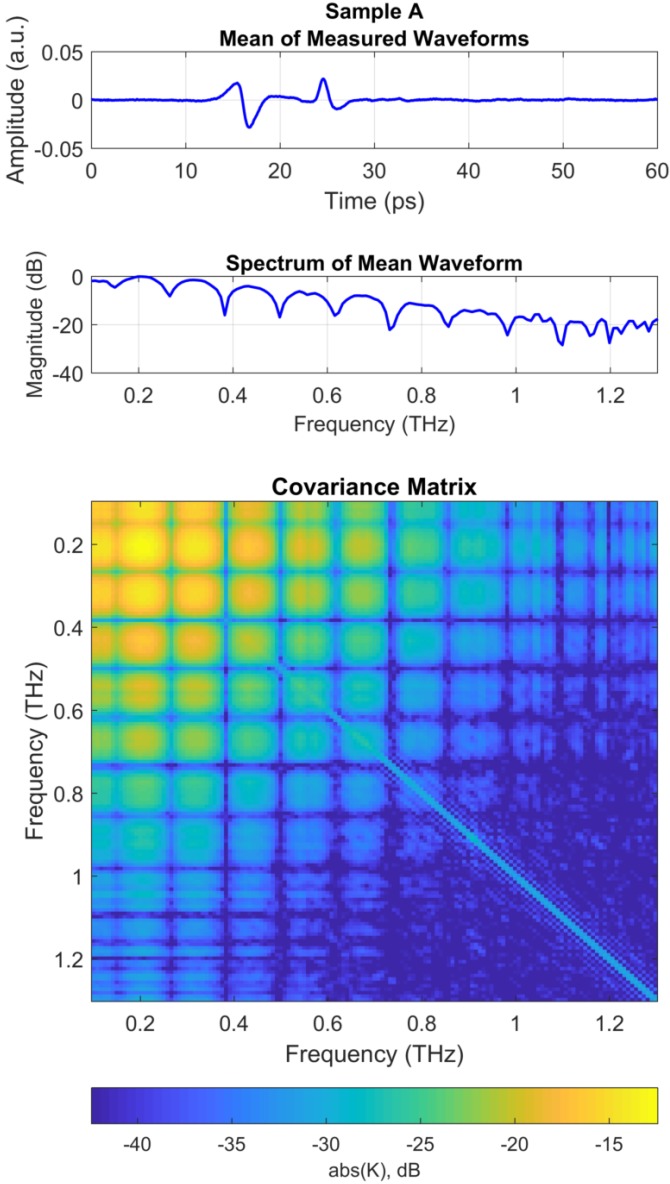
Measured data for Sample A. (**Top**) Mean of measured THz waveforms. Note that reflected THz pulse 1 (illustrated in Figure 1) arrives at approximately 15 ps, and pulses 2 and 3 overlap with one another at approximately 25 ps; (**Middle**) Spectrum of mean waveform in the top panel; (**Bottom**) Covariance matrix computed with Equation (7) using the spectrum of each 300 measured waveform.

The top panel of Figure 2 shows the mean THz waveform for Sample A. Note that reflected THz pulse from the top surface of polycarbonate layer arrives at approximately 16 ps. The THz pulses from the upper and lower boundaries of the air gap overlap with one another resulting in a single peak at approximately 24.5 ps. Thus, thickness of $d_{2}$ layer cannot be estimated from a simple TDOA analysis based on impulse response or matched filter analysis as discussed in Section 3.2. The middle panel of Figure 2 shows the spectrum of the mean waveform in the top panel. The spectrum was truncated to include the band with maximum SNR, i.e., 0.1–1.3 THz. Note that the spectrum shows peaks and nulls separated by approximately 116.5 GHz, due to the multipath delay of approximately $1/(1.165\times 10^{11})=8.5$ ps between the THz pulse from the polycarbonate surface and the THz pulses from the air gap.

An FFT was performed on the each of the truncated THz waveforms and the 300 resulting spectra were truncated within the bandwidth of 0.1–1.3 THz for maximum SNR. Each of the spectra were normalized to unit vectors, as discussed in Section 2. This normalization is not necessary for MFP, but provides a maximum possible output of unity in the Bartlett processor if the replica weight vectors are also normalized to unit vectors [24,29]. The covariance was then estimated using Equation (7) with L=145 frequency bins and Z=300 snapshots. The resulting covariance matrix is a L×L square matrix of complex values. The bottom panel of Figure 2 shows the absolute value of the covariance matrix on a dB scale.

Several features of the covariance matrix in Figure 2 can be observed. Higher levels of covariance appear for the covariance between low frequency bins in the upper left corner of the covariance matrix due to the higher spectral levels at low frequencies of the measured THz spectra. Conversely, the lower right corner of the covariance matrix shows relatively low covariance between high frequency bins because of the lower spectral levels at the high end of the THz spectrum. Stronger covariance is observed along the diagonal of the covariance matrix due to the high level of auto-covariance for each frequency bin, relative to adjacent bins. The covariance matrix is symmetric about the diagonal because the magnitude of the covariance between each pair of frequency bins is the same. Finally, we note that the peaks and nulls observed in the THz spectrum (middle panel of Figure 2) are also evident at 116.5 GHz intervals in the covariance matrix due to the high level of covariance around the peaks in the spectra.

**Figure 3:**
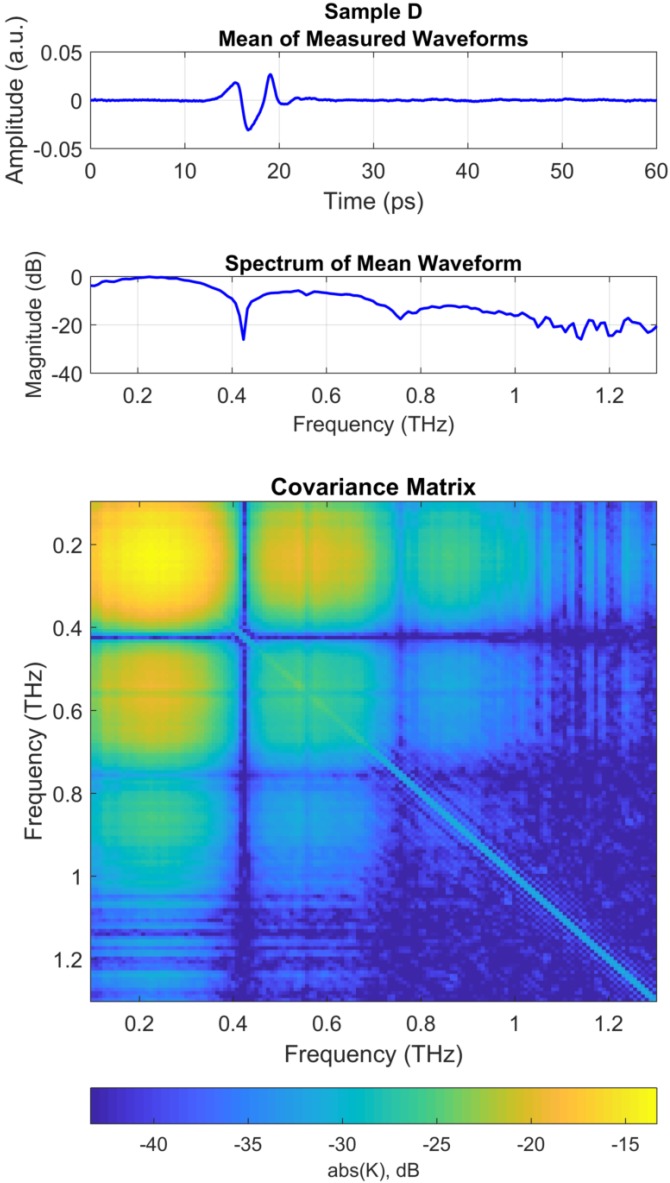
Measured data for Sample D. (**Top**) Mean of measured THz waveforms. Note that reflected THz pulses 1, 2 and 3 (illustrated in Figure 1) all overlap on one another; (**Middle**) Spectrum of mean waveform in the top panel; (**Bottom**) Covariance matrix computed with Equation (7) using the spectrum of each 300 measured waveform.

Data were processed for the other samples (Samples B–D) in a manner similar to the method discussed for Sample A above. Figure 3 shows the processing results for Sample D, which had the thinnest surface layer, $d_{1}$, of all the samples.

The top panel of Figure 3 shows the mean THz waveform for Sample D, where the reflected THz pulse from the top surface of polycarbonate layer arrives at approximately 16 ps. However, due to the thin layer of polycarbonate in Sample D, the THz pulses from the lower surface of the polycarbonate layer overlaps with the THz pulses reflected from the upper and lower boundaries of the air gap resulting in a single distorted waveform between 13 and 21 ps. Thus, thickness of layers $d_{1}$ and $d_{2}$ cannot be estimated from a simple TDOA analysis. The following section demonstrates that the THz MFP approach discussed in Section 2 can be used to accurately estimate the thickness of both of these layers.

The middle panel of Figure 3 shows the spectrum of the mean waveform in the top panel, truncated with the bandwidth of 0.1–1.3 THz. The spectrum shows peaks and nulls separated by approximately 333 GHz, due to the multipath delay of approximately $1/(3.33\times 10^{11})=3$ ps between the THz pulse from the polycarbonate surface arriving at 16 ps and the subsequent overlapping THz pulses arriving at 19 ps.

The covariance matrix for Sample D was generated in a manner similar to that used for Sample A. The bottom panel of Figure 3 shows the absolute value of the covariance matrix for Sample D. Note that the covariance matrix shows similar features to that of the covariance matrix for Sample A (bottom panel of Figure 2) except that peaks and nulls are observed at intervals of approximately 333 GHz, due to the shorter delay time between the arrival of multipath pulses in this sample as discussed above.

### 3.4. Generating Replica Spectra

As discussed in Section 2, the MFP approach compares the covariance matrix with a set of modeled replica fields. The transfer matrix model discussed in Section 2 was used to generate a set of transfer functions, $H_{s}\left(f,a\right)$, for the layered media sample illustrated in Figure 1, where the parameter vector, a, contained the thicknesses of the three layers, i.e., $a={\left[d_{0},d_{1},d_{2}\right]}^{T}.$ A range of possible thicknesses in were considered for each of the three layers. A total of 51 thicknesses from 0–100 μm in 10 μm intervals were considered for the calibration layer, $d_{0}$, and a total of 101 thicknesses from 0 to 1000 μm in 10 μm intervals were considered for both the polycarbonate layer, $d_{1}$, and air gap layer, $d_{2}$. Thus, a total of 51×101×101 = 520,251 replicas were computed and compared with the measured data.

Prototype code was developed in Matlab to perform the MFP computations on an Intel Xeon 2.80 GHz processor with 64 GB of random access memory (RAM). All of the replica spectra were generated in approximately two hours, and then saved to a file. The resulting set of replicas could then be used for MFP with each of the THz sample measurements (Samples A–D) within a few minutes for each sample. It is expected that more efficient code could be developed for faster processing in the future.

The index of refraction Equation (A4) used for the calibration layer and the air gap between the polycarbonate layers was n=1 for all frequencies. Thus, the absorption spectra of the air was neglected because it is expected to have negligible impact on the measured spectra for these thin layers with thicknesses on the order of a few hundred microns. The frequency-dependent index of refraction for the polycarbonate layers was taken from a separate transmission measurement, as discussed in Section 3.2.

### 3.5. THz MFP Results for Sample A

The covariance matrix in Figure 2 was compared with the replica weight vectors (discussed in Section 3.4) using the Bartlett processor and the MV processor using Equations (9) and (10), respectively. The output, P(a), of each processor is a scalar value for each possible parameter vector, a. Thus, parameter vector corresponding to the maximum value of the processor output is taken to be the the best estimate, $\hat{a}$, for the unknown parameters.

For Sample A, the best estimate of the parameter vector from the Bartlett processor was ${\hat{a}}_{B}={\left[190,750,70\right]}^{T}$ μm, and the best estimate of the parameter vector from the MV processor was ${\hat{a}}_{\mathrm{MV}}={\left[180,750,70\right]}^{T}$ μm. Thus, the estimated thickness of the calibration layer was nearly the same for both processors and accurate to within the measurement resolution of the Vernier caliper and the replicas that were generated (10 μm).

**Figure 4:**
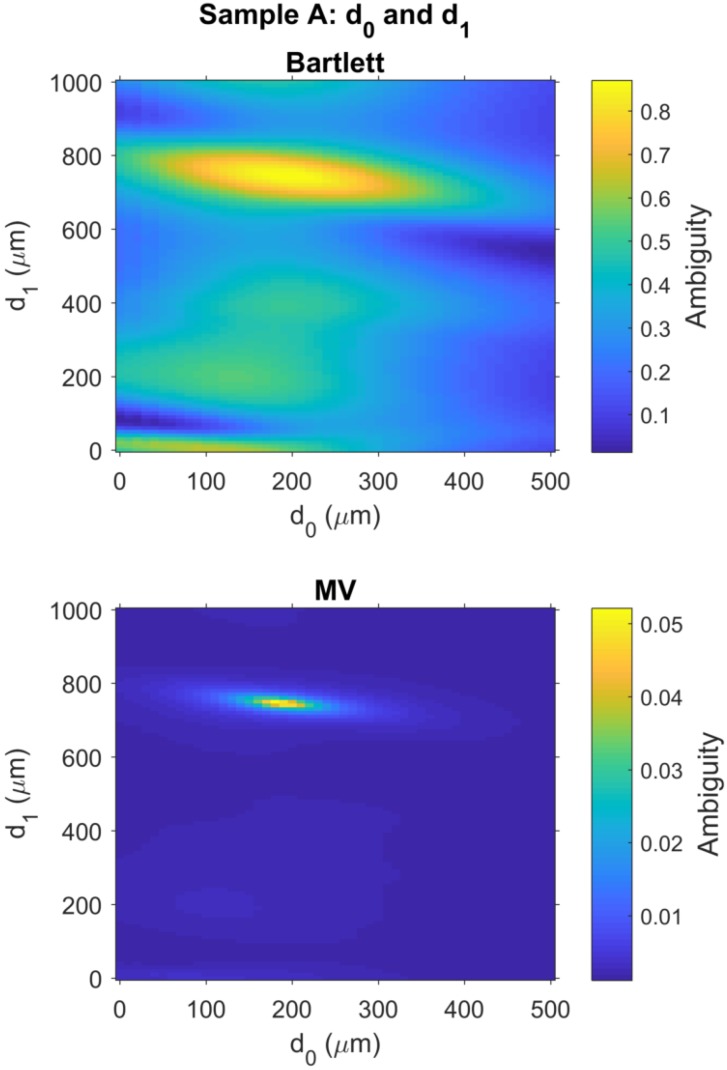
Matched field ambiguity surfaces provide estimates for the thickness of the calibration layer, $d_{0}$, and polycarbonate layer, $d_{1}$, as illustrated in Figure 1 with layer thickness for Sample A given in Table 1. (**Top**) Bartlett processor has a global maximum $d_{0}=190$ μm and $d_{1}=750$ μm; (**Bottom**) MV processor has a global maximum $d_{0}=180$ μm and $d_{1}=750$ μm. The results for $d_{1}$ are consistent with ground truth measurements with a Vernier caliper. Ground truth data are not available for the calibration layer, but the these results are reasonable, and consistent between both Bartlett and MV processors. See Table 2 for a comparison of measurement errors for layers in all samples.

As discussed in Section 2.6, ambiguity surfaces are often used in the MFP literature to evaluate the ambiguity around the estimated parameters. For Sample A, a 2D image of the ambiguity surface for the Bartlett processor output was generated for $d_{0}$ vs. $d_{1}$ by selecting the ambiguity data for which the $d_{2}={\hat{d}}_{2,B}$ and organizing the remaining ambiguity data into the 2D image shown in the top panel of Figure 4. The ambiguity surface for the Bartlett processor in the top panel of Figure 4 shows relatively high ambiguity surrounding the global maximum, located at coordinates ${\hat{d}}_{0,B}=190$ μm and ${\hat{d}}_{1,B}=750$ μm.

Similarly, for Sample A, a 2D image of the ambiguity surface for the MV processor output was generated for $d_{0}$ vs. $d_{1}$, as shown in the bottom panel of Figure 4. As noted in Section 2.5.2, the MV processor uses adaptive processing to suppress ambiguities around the best estimate. As a result, a very narrow peak is observed in the ambiguity surface surrounding the global maximum, located at coordinates ${\hat{d}}_{0,MV}=180$ μm and ${\hat{d}}_{1,MV}=750$ μm.

**Figure 5:**
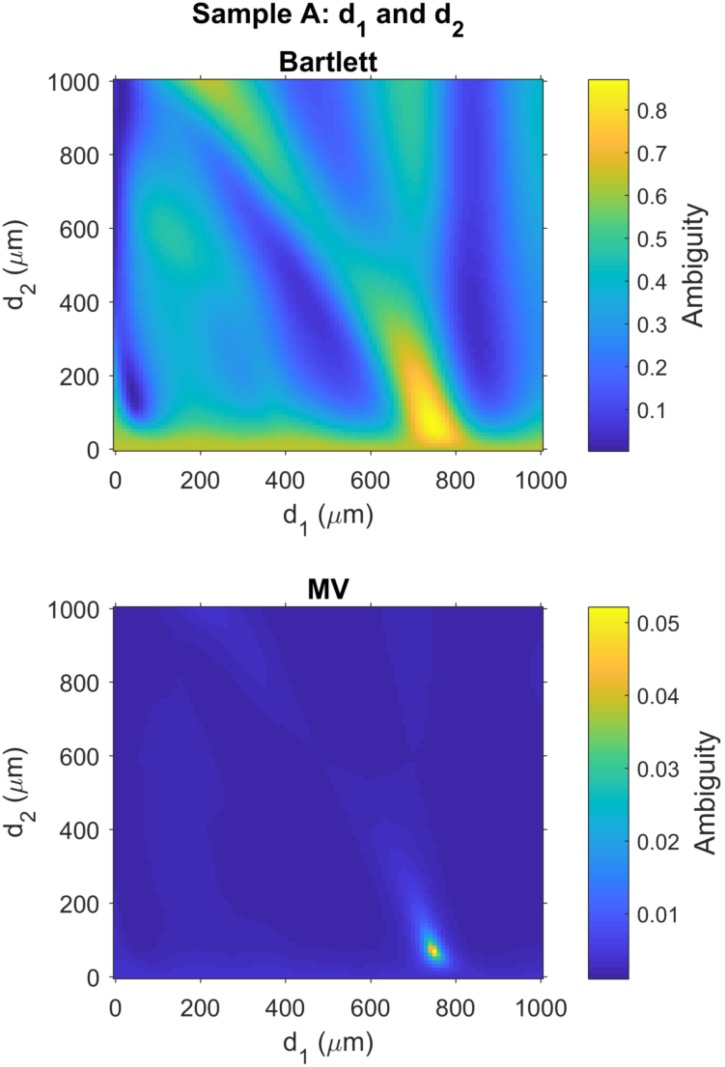
Matched field ambiguity surfaces provide estimates for the thickness of the polycarbonate layer, $d_{1}$ and the air gap, $d_{2}$, as illustrated in Figure 1 with layer thickness for Sample A given in Table 1. (**Top**) Bartlett processor has a global maximum $d_{1}=750$ μm and $d_{2}=70$ μm; (**Bottom**) MV processor has a global maximum $d_{1}=750$ μm and $d_{2}=70$ μm. These results are consistent with ground truth measurements with a Vernier caliper. See Table 2 for a comparison of measurement errors for layers in all samples.

Next, for Sample A, the ambiguity surface for $d_{1}$ vs. $d_{2}$ was created for the Bartlett processor by selecting the ambiguity data from the Bartlett processor output for which the $d_{0}={\hat{d}}_{0,B}$ and then the remaining ambiguity data was organized into the 2D image shown in the top panel of Figure 5. A similar process was used to create the ambiguity surface for the MV processor output shown in the bottom panel of Figure 5. The ambiguity surface for the Bartlett processor shows a relatively broad peak surround the global maximum located at coordinates ${\hat{d}}_{1,MV}=750$ μm and ${\hat{d}}_{2,B}=70$ μm. As noted above, the MV processor uses adaptive processing to reduce ambiguities around the global maximum, which was also located at coordinates ${\hat{d}}_{1,MV}=750$ μm and ${\hat{d}}_{2,MV}=70$ μm.

### 3.6. THz MFP Results for Sample D

Similar to the analysis for Sample A in Section 3.5, the covariance matrix in Figure 3 was compared with the replica weight vectors using the Bartlett processor and the MV processors.

For Sample D, the best estimate of the parameter vector from the Bartlett processor was ${\hat{a}}_{B}={\left[180,250,60\right]}^{T}$ μm, and the best estimate of the parameter vector from the MV processor was ${\hat{a}}_{\mathrm{MV}}={\left[190,240,60\right]}^{T}$ μm. Thus, the estimated thickness for each of the layers was within only 10 μm of the thicknesses measured for these layers with a digital Vernier caliper. A detailed error analysis for Samples A–D is provided at the end of this section.

**Figure 6:**
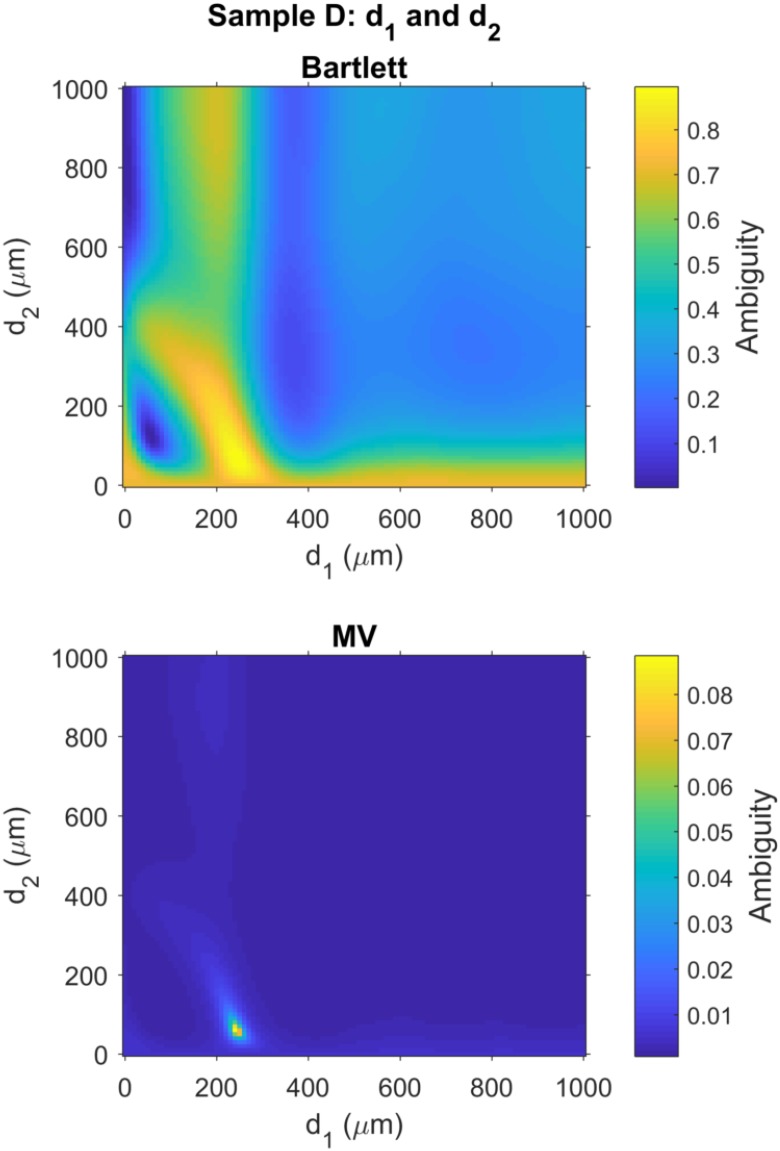
Matched field ambiguity surfaces provide estimates for the thickness of the polycarbonate layer, $d_{1}$, and the air gap, $d_{2}$, as illustrated in Figure 1 with layer thickness for Sample D given in Table 1. (**Top**) Bartlett processor has a global maximum $d_{1}=250$ μm and $d_{2}=60$ μm; (**Bottom**) MV processor has a global maximum $d_{1}=240$ μm and $d_{2}=60$ μm. These results are consistent with ground truth measurements with a Vernier caliper. See Table 2 for a comparison of measurement errors for layers in all samples.

Finally, ambiguity surfaces for Sample D were generated using techniques identical to those used for the ambiguity surface images presented for Sample A, above. The top and bottom panels of Figure 6 show the ambiguity surfaces for Bartlett and MV processors, respectively, for layers $d_{1}$ and $d_{2}$. The ambiguity surface for the Bartlett processor shows a relatively broad peak surround the global maximum located at coordinates ${\hat{d}}_{1,MV}=250$ μm and ${\hat{d}}_{2,B}=60$ μm with high ambiguity also spread along the bottom edge of the ambiguity surface, i.e., small values of $d_{2}$. As discussed for Sample A, the MV processor also shows reduced ambiguities around the global maximum for Sample D, located at coordinates ${\hat{d}}_{1,MV}=240$ μm and ${\hat{d}}_{2,MV}=60$ μm.

### 3.7. Error Analysis for All THz MFP Results

The same measurement techniques and signal processing steps discussed in Section 3.5 and Section 3.6 were applied to estimate the thicknesses of each of layers shown in Figure 1 for each of the samples (Samples A–D) listed in Table 1, and an error analysis was performed to evaluate the performance of the THz MFP approach with a conventional measurement.

Ambiguity surfaces (not shown here) were created for each of the layers in each of the samples. In each case, the global maximum of the Bartlett and MV ambiguity surfaces appeared at approximately the same depth coordinates as discussed above, and the MV processor suppressed ambiguities around the global maximum, similar to the ambiguity surface results plotted in Figure 4, Figure 5 and Figure 6. The best estimate of the thicknesses from the Bartlett and MV processors are recorded in Table 2, along with thickness measurements from a digital Vernier caliper. Note, Vernier caliper measurements are not available for comparison with the calibration layer.

The same measurement resolution was used for the Vernier caliper and the THz MFP thickness estimates. The measurement resolution and accuracy of the Vernier caliper were 10 μm and 20 μm, respectively. Similarly, the thickness interval between each of the trial thicknesses that were used to create the replicas was 10 μm. The results of the thickness estimates from THz MFP are in excellent agreement with the measurements from the Vernier caliper. All of the differences between the THz MFP results and the Vernier caliper measurements are within the measurement resolution of the Vernier caliper.

**Figure 7:**
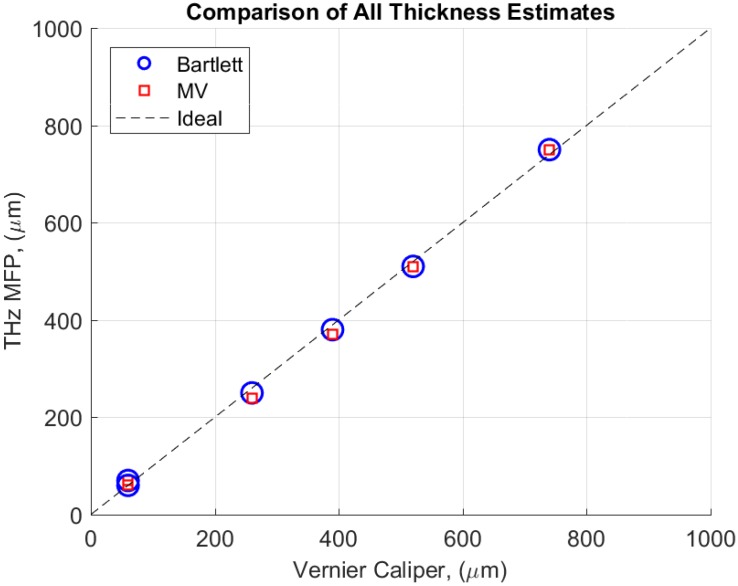
For each of the Vernier caliper measurements listed in Table 2, the corresponding thickness estimates from THz MFP with the Bartlett and MV processors are plotted as blue circles and red squares, respectively. The dashed line represents an ideal case of equality of the Vernier caliper measurement and the THz MFP thickness estimate. Thus, all of the measurement data from the THz MFP approach is in close agreement with the Vernier caliper data.

A visual comparison of the thickness data listed is Table 2 is provided in Figure 7. For each of the Vernier caliper measurements listed in the table, the corresponding thickness estimates from THz MFP with the Bartlett and MV processors are plotted as blue circles and red squares, respectively. The dashed line represents an ideal case of equality of the Vernier caliper measurement and the THz MFP thickness estimate. Thus, all of the measurement data from the THz MFP approach is in close agreement with the Vernier caliper data.

## 4. Conclusions

The authors applied MFP techniques that have been developed over several decades for sonar and remote sensing applications in noisy and dynamically changing environments. A mathematical model for the THz field was developed and the methods to compare these modeled spectra with the covariance matrix of the measured THz field were outlined. The THz MFP techniques were then applied to real THz data that were collected with a THz-TDS system for a variety of layer thicknesses. The THz MFP techniques presented here were used to simultaneously estimate the thickness of multiple layers within the measurement accuracy of the Vernier caliper used for ground truth measurements.

The samples considered here were representative of NDE applications in which it is desirable to know the thickness of a defect (e.g., air bubble) and its depth below the surface. The thickness of a thin air gap and the thickness of the polycarbonate layer above it were both determined as shown in Figure 5 and Figure 6 and summarized in Table 2. Thus, MFP was used to simultaneously determine layer thicknesses for both polycarbonate, $d_{1}$, and an air gap, $d_{2}$, (see Figure 1) for the four polycarbonate layer thicknesses (A, B, C and D) listed in Table 1. In addition, the thickness of the calibration layer, $d_{0}$, was also determined in each case, which is only needed for alignment purposes as explained in Section 3.2.

Here, the measurement configuration was oriented for monostatic measurement configuration at normal incidence. However, a similar methodology could potentially be applied to a bi-static configuration with the receiver oriented to measure the specular reflection of a THz pulse at oblique incidence. This is accounted for with the angle of incidence, $\phi_{iq}$, in Equation (A5) in the propagation model discussed in Appendix A.

The ambiguity surfaces illustrate the performance of two of the most popular objective functions in the MFP literature: the Bartlett processor and the MV processor. These results demonstrated the potential of both the Bartlett and MV processors to accurately estimate the thickness of thin films as well as thick layers from real THz data collected with a pulsed THz-TDS system and a propagation model that can account for reflections with multiple co-planar layers. As expected, the MV processor suppresses more of the ambiguities that surround the global maximum as compared to the Bartlett processor.

The techniques demonstrated here can be applied to THz NDE applications in which the materials within each layer are known and the primary goal of the model-based parameter estimation is to indirectly measure the layer thicknesses. If the complex index of refraction is not well-known a priori, then it may be parameterized and included as a set of free variables in the parameter vector.

The techniques presented here could also be extended to THz synthetic aperture arrays using mathematical approaches in the MFP literature [29]. THz synthetic aperture imaging can be performed by raster scanning a monostatic THz emitter/sensor pair above the sample under test [30,31,32,33].

To the author’s knowledge, this is the first time that the Bartlett or MV processors have been applied for THz NDE of thin films, and that ambiguity images have been used to compare the performance of objective functions for THz-TDS data. It is expected that this research will provide an important link for THz researchers to access and apply the robust methods available in the MFP literature.

## Figures and Tables

**Table 1:** Four samples of thin polycarbonate films (layer $d_{1}$ in Figure 1) were evaluated in this study. Each of the samples was measured with a digital Vernier caliper. The mean of 10 measurements for each sample film is listed in the table.

Sample	Manuf. Spec. (mils)	Vernier Cal. (μm)
A	30 mil	740
B	20 mil	520
C	15 mil	390
D	10 mil	260

**Table 2:** Thickness estimates obtained from THz MFP with the Bartlett and MV objective functions for the experiment configuration shown in Figure 1. All of the differences between the THz MFP results and the Vernier caliper measurements are within the measurement resolution of the Vernier caliper (20 μm).

Sample	Layer	Vernier Cal.	THz MFP Bartlett	THz MFP: MV
ID	ID	(μm)	(μm)	(μm)
A	$d_{1}$	740	750	750
A	$d_{2}$	60	70	70
B	$d_{1}$	520	510	510
B	$d_{2}$	60	70	70
C	$d_{1}$	390	380	370
C	$d_{2}$	60	60	70
D	$d_{1}$	260	250	240
D	$d_{2}$	60	60	60

## Appendix A. Propagation within Layered Media

The MFP algorithm requires a forward propagation model to generate replicas of the THz spectrum that can be later compared with the measured THz spectrum by an objective function. This appendix describes the propagation model used to simulate the sample’s transfer function, $H_{s}\left(f\right)$. Parameterization of the model in terms of the parameter vector, a, is discussed at the end of this section.

Many samples of interest (e.g., laminated composites, painted surfaces, etc.) can be approximated as a stack of plane-parallel layers. If the sample is within the focal depth of both the transmit and receive lenses of the THz TDS system, then the propagation of the THz waves may be modeled as plane waves propagating within the stack of layers.

The composite reflection or transmission coefficient for a parallel stack of an total number of *Q* layers can be computed efficiently using a matrix model [34]. Thus, the sample’s transfer function, $H_{s}\left(f\right)$, for the THz measurement with the sample composed of plane parallel layers can be expressed as [34]

(A1) $$H_{s}\left(f\right)=\frac{Y_{0}m_{11}+Y_{0}Y_{s}m_{12}-m_{21}-Y_{s}m_{22}}{Y_{0}m_{11}+Y_{0}Y_{s}m_{12}+m_{12}+Y_{s}m_{22}},$$

if the THz-TDS system is configured in *reflection* mode. A similar expression can be used if the sample is measured in transmission mode [34].

In Equation (A1), $Y_{0}$ and $Y_{s}$ are the admittances of the semi-infinite media above and below the layer stack, respectively. The specific details and formulas for computing the admittance values are available in [34]. In Equation (A1), $m_{11},m_{12},m_{21}$ and $m_{22}$ are the four elements of a 2×2 matrix, M, given by

(A2) $$M=M_{1}\times M_{2}\ldots \times M_{q}\times \ldots \times M_{Q}=\left[\begin{array}{cc} m_{11} & m_{12}\\ m_{21} & m_{22} \end{array}\right],$$

where the matrices $M_{1},M_{2},\ldots M_{q}\ldots M_{Q}$ each relate the electric and magnetic fields at the boundaries of layers 1,2…q…Q, respectively. The matrix for an arbitrary layer, *q*, is computed as [34],

(A3) $$M_{q}=\left[\begin{array}{cc} cos(k_{0}{\tilde{n}}_{q}h_{q}) & jsin\left(k_{0}{\tilde{n}}_{q}h_{q}\right)/Y_{q}\\ jY_{q}sin\left(k_{0}{\tilde{n}}_{q}h_{q}\right) & cos(k_{0}{\tilde{n}}_{q}h_{q}) \end{array}\right],$$

where $j=\sqrt{-1}$, $Y_{q}$ is the admittance of the medium in layer *q*; $k_{0}=\left(2\pi f\right)/c$ is the wave number in free space; and ${\tilde{n}}_{q}$ is the *complex* index of refraction for layer *q*. The complex index of refraction for each layer, ${\tilde{n}}_{q}$, is frequency-dependent and can be expressed as

(A4) $${\tilde{n}}_{q}\left(f\right)=n_{q}\left(f\right)+j\kappa_{q}\left(f\right).$$

The real part of the refractive index, $n_{q}\left(f\right)$, accounts for dispersion and the extinction coefficient, $\kappa_{q}\left(f\right)$, accounts for attenuation within the layer, *q*. The signal processing methods for determining the complex index of refraction of a sample material from an independent THz TDS measurement are already documented in the literature [2,37] and therefore are not discussed here.

In Equation (A3), $h_{q}$ is the propagation distance inside layer *q* given by,

(A5) $$h_{q}=d_{q}cos\left(\phi_{iq}\right).$$

where $\phi_{iq}$ is the angle of propagation within the layer, *q*, relative to the surface normal, due to Snell’s law, and $d_{q}$ is the thickness of layer *q*. Thus, for normal incidence, Equation (A5) simplifies to $h_{q}=d_{q}$.
